# Galactagogue activity of shalukam (*Nelumbium speciosum* Willd rhizome) compared to domperidone in Wistar albino rats

**DOI:** 10.3389/fphar.2026.1810350

**Published:** 2026-06-08

**Authors:** Aathira S. Kumar, K. S. Vimala, S. Priya, B. Priyalatha

**Affiliations:** Department of Dravyaguna Vigyana, Amrita School of Ayurveda, Amritapuri, Amrita Vishwa Vidyapeetham, Kollam, India

**Keywords:** domperidone, galactagogue activity, *in vivo*, Nelumbium speciosum rhizome, shalukam

## Abstract

Breast milk insufficiency is a significant public health concern, as it directly affects infant nutrition and immunity. Inadequate milk production remains a major barrier to effective breastfeeding, and it is reported in approximately 60 -90% of mothers in low- and middle-income countries. Emotional stress, anxiety, lack of sleep, and maternal illness are various psychological and physiological factors that influence breast milk production. Galactagogues are used to induce, maintain, and increase breast milk production. Commonly used galactagogues include domperidone, metoclopramide, chlorpromazine, and sulpiride. These medications are associated with side effects such as xerostomia, gastrointestinal disorders, cardiac arrhythmia, lethargy, sedation, and hypertension. In Ayurvedic classical texts, several drugs possessing sthanya janana properties are described. *Bhavaprakasha Nighantu* and *Priya Nighantu* mention *Śālūkaṃ* as *sthanya pradam*. In an article titled “Report on folklore medicinal plants used for female healthcare in Assam”, a tablet prepared from the rhizome of Nelumbium speciosum Willd. is reported to be used to increase lactation and to manage painful menstruation. Twenty- four female Wistar albino rats were divided into four groups, with six rats in each group. Two trial drug groups received *śālūkaṃ chūrṇa* (finely powdered dried plant material) at single and double doses, while the control group received standard feed, and the standard group received domperidone from the 5th to the 14th day of lactation. All treatments were administered orally in distilled water using an oral gavage needle at 1:30 PM daily. The *chūrṇa* of *śālūkaṃ* is administered by mixing 1.08g/kg of in 10ml distilled water in Single dose group, 2.16g/kg in 20ml distilled water and 1g/kg of Domperidone in 10ml distilled water in standard group. Milk yield was estimated by separating the mothers from the pups for four hours, followed by 60 minutes of suckling, with pre- and post-suckling body weights measured using an electronic balance. The results suggest that the trial drug double-dose group demonstrated better galactagogue activity than the standard drug domperidone group in parameters serum prolactin and cortisol levels and trial drug single dose group demonstrated better galactagogue activity than the standard drug domperidone group in parameters serum prolactin and milk yield of 10 days. The findings suggests that the double dose group demonstrates stronger galactagogue potential.

## 1 Introduction

Breast milk insufficiency is a significant public health concern, as it directly affects infant nutrition and immunity. Inadequate milk production remains a major barrier to successful breastfeeding and has been reported in approximately 60%–90% of mothers in low- and middle-income countries (Piccolo et al., 2022). Psychological and physiological factors such as emotional stress, anxiety, sleep deprivation, and maternal illness can adversely influence breast milk production (Lajuna and Sriyanti, 2025). Galactagogues are synthetic or plant-derived substances used to initiate, maintain, or enhance breast milk secretion (Machado et al., 2014). In modern medicine, commonly used galactagogues include domperidone, metoclopramide, chlorpromazine, and sulpiride. However, these drugs are associated with adverse effects such as xerostomia, gastrointestinal disturbances, cardiac arrhythmias, lethargy, sedation, and hypertension in breastfeeding mothers (Murthy, 2005; Bora et al., 2016).

Ayurvedic classical texts describe numerous drugs possessing *stanya janana* (lactation-promoting) properties. *Bhavaprakasha Nighantu* and *Priya Nighantu* mention *śālūkaṃ* (*Nelumbium speciosum* Willd) as *stanya pradāna* (galactagogue). Traditionally, *śālūkaṃ* has been administered internally for conditions such as *mūtravrodha* (urinary retention), *śukradraṃs.ṭrodbhava jvara*, and *krimidanta* (worm infestation).

An article titled “Report on Folklore Medicinal Plants Used for Female Healthcare in Assam” reports the use of tablets prepared from the rhizome of *N. speciosum* Willd to enhance lactation and manage painful menstruation. Additionally, its use in the management of liver and heart disorders, dysentery, dyspepsia, vomiting, bleeding piles, and menorrhagia has been documented in an article titled “Plants of Medicinal Importance from Halia Block of District Mirzapur, Uttar Pradesh” (Tiwari and Singh, 2012).

The rhizome of *N. speciosum* Willd contains phytoconstituents such as flavonoids, rutin, quercetin, kaempferol, and phenolic compounds, which have demonstrated supportive galactagogue activity in other medicinal plants and have been experimentally validated for lactation-enhancing effects (Kumar and Gupta, 2015; Lin et al., 2018; Mukherjee et al., 2009).

Although numerous *stanya janana* drugs are described in Ayurvedic literature, the identification of alternative sources is essential to prevent overexploitation of traditionally used plants. The therapeutic potential of *N. speciosum* Willd rhizome remains relatively unexplored. Furthermore, future comparative studies on the galactagogue activity of plants possessing *stanya janana* properties may help identify species with superior lactation-promoting potential. Therefore, the present study was undertaken to evaluate the galactagogue activity of *śālūkaṃ* (*N. speciosum* Willd rhizome) in Wistar albino rats.

## 2 Materials and methods

### 2.1 Identification and collection of the trial drug

The trial drug, the rhizome of *N. speciosum* Willd, was taxonomically identified. A herbarium specimen of the plant was deposited at the Jawaharlal Nehru Tropical Botanic Garden and Research Institute, Palode, Thiruvananthapuram, Kerala, India.

### 2.2 Pharmacognostical, physicochemical, and phytochemical analysis of the trial drug

#### 2.2.1 Study centers

Pharmacognostical, phytochemical, HPTLC, and heavy metal analyses were conducted at CARe KERALAM Limited, KINFERA Park, Koratty, Kerala. Physicochemical analysis was carried out at the Quality Control Laboratory, Amrita School of Ayurveda, Kollam, Kerala.

#### 2.2.2 Methods

Macroscopic and microscopic evaluation, physicochemical analysis, phytochemical analysis, HPTLC, and heavy metal analysis of the trial drug were conducted.

##### 2.2.2.1 HPTLC

1 g of *śālūkaṃ* (*N. speciosum* Willd) rhizome powder was suspended in 10 mL of Methanol. 6, 6 and 6 µL of the above extract was applied on a pre-coated silica gel F254 on aluminium plates to a band length of 8 mm using Linomat 5 TLC applicator. The plate was developed in chloroform: Ethylacetate: formic acid (5:4:0.5). The developed plates were visualized under short UV, long UV and then derivatised with Anisaldehyde-sulphuric acid and scanned under UV 254 nm, 366 nm and 620 nm. Rf, colour of the spots was recorded.

### 2.3 Toxicity

The acute toxicity assessment conducted in earlier studies indicated that the LD_50_ of both aqueous and methanolic extracts of *N. speciosum* rhizome exceeded 5,000 mg/kg, suggesting that the extracts are safe for use (Rahman et al., 2019).

## 3 *In vivo* study

### 3.1 Study centre

Department of Toxicology, SDM Centre for Research in Allied Sciences, Udupi, Karnataka, India.

### 3.2 Ethical clearance statement

Institutional Animal Ethics Committee (IAEC) approval was obtained from the Institutional Ethics Committee of the SDM Centre for Research in Ayurveda and Allied Sciences, Udupi, Karnataka, India (Approval No. IAEC No: SDMCRA/IAEC/AM-13-2025).

### 3.3 Aim and objectives

The present study aimed to evaluate the galactagogue activity of *śālūkaṃ* (*N. speciosum* Willd rhizome) in Wistar albino rats.

### 3.4 Animal selection criteria

Healthy female Wistar albino rats (n = 24), weighing approximately 250–300 g, were maintained under standard laboratory conditions with controlled temperature and a light–dark cycle.

### 3.5 Inclusion criteria

Healthy female Wistar albino rats weighing 250–300 g was included in the study.

### 3.6 Exclusion criteria

- Rats previously subjected to other experimental procedures
- Rats with pathological conditions.

### 3.7 Grouping

The 24 female Wistar albino rats were divided into four groups of six rats each (Badgujar and Bandivdekar, 2015). The groups were designated as the control group, standard group, *śālūkaṃ* trial drug single dose group, and *śālūkaṃ* trial drug double-dose group (Table 1).

**TABLE 1:** The experiment protocol for galactagouge activity.

SI. no	Name of the groups	Drug	Number of animals	Route of administration	Dose
1	Control group	Distilled water	6	Oral	—
2	Standard group	Domperidone	6	Oral	1 g/kg Domperidone + 10 mL distilled water
3	Trial drug single dose group	Churna of *śālūkaṃ*	6	Oral	1.080 g/kg churna +10 mL distilled water
4	Trial drug double dose group	Churna of śālūkaṃ	6	Oral	2.16 g/kg churna + 20 mL distilled water

### 3.8 Sample size justification

The sample size for the present study was determined based on standard practices in experimental animal research, taking into account both statistical reliability and ethical considerations. A total of 24 rats were allocated into four groups with six animals in each group (n = 6), which is commonly considered adequate for detecting meaningful biological differences in pharmacological studies. Previous methodological studies have indicated that a group size of 5–6 animals is generally sufficient to achieve acceptable statistical power when moderate to large treatment effects are expected and data are analyzed using appropriate statistical tests such as one-way ANOVA followed by appropriate *post hoc* tests (Charan and Kantharia, 2013). In addition, established guidelines emphasize minimizing animal use while maintaining scientific validity, supporting the use of small, well-designed experimental groups (Festing and Altman, 2002). Therefore, the selected sample size was considered appropriate for the objectives of the study.

### 3.9 Dose fixation

Animal dose was calculated based on body surface area ratio and the suitable rats dose using the Paget & Barner’s rule -1964.

Animal dose = Human dose x conversion factor (0.018) for rat × 5

Human dose = 12 g Dose of Churna kalpana (Samhita and Murthy, 2012).

#### 3.9.1 Rat dosage

Trial drug- *śālūkaṃ* (*N. speciosum* Rhizome churna)

Human dose=12 gm

Rat dose=12×0.018×5

=1.080g/kg

=1080mg

Trial drug double dose- *śālūkaṃ* (*N. speciosum* Rhizome churna)

Human dose=12 gm

Rat dose=2×12×0.018×5

=2.160g/kg

=2160mg

Standard drug (Domperidone)

Rat dose=2.5g/kg

=0.0025mg/g

### 3.10 Experimental procedure

Twenty-four female rats and twelve male rats were randomly selected from the animal house. Two female rats and one male rat were housed together for 21 days. The body weight of the female rats was recorded on the first day and subsequently on a weekly basis to confirm pregnancy. After confirmation of pregnancy, the male rats were removed from the cages. The day of delivery was considered day one of lactation. All lactating rats were randomly divided into four groups, with six rats in each group (n = 6). Each dam was standardized to six pups.

Two trial groups received *śālūkaṃ* (*N. speciosum* Willd rhizome) chūrṇa at single and double doses, respectively. The control group received a standard diet, and the standard group received domperidone. Treatments were administered orally from the 5th to the 14th day of lactation using distilled water as the vehicle using an oral gavage needle at 1:30 p.m. daily. The chūrṇa of *śālūkaṃ* is administered by mixing 1.08 g/kg of chūrṇa in 10 mL distilled water in Single dose group, 2.16 g/kg in 20 mL distilled water and 1 g/kg of Domperidone in 10 mL distilled water in standard group.

On 5th day at 8.30 a.m. pups and dams were separated. At 12.30 p.m. weight of the pups and dam were taken and kept together for suckling. At 1.30 p.m. the weight of the pups and the dams were taken again. The pups weight were calculated by subtracting initial pups weight from final pups weight.

Milk yield was estimated by separating the dams from their pups for 4 h, followed by 60 min of suckling. Pup weights were recorded before and after suckling using an electronic balance with an accuracy of 0.1 g. Milk yield was measured daily from 5th to 14th day of lactation (Sampson and Jansen, 1984; Hosseinzadeh et al., 2013). The mean initial weight of pups over the 10-day period was calculated by summing the daily initial weights recorded from day 5 to day 14 of lactation and dividing the total by 10. Similarly, the mean final weight of pups was calculated by summing the corresponding daily final weights over the same period and dividing by 10.

On the 15th day, rats were anesthetized by administering 10 mL of 3% Isoflurane as inhalation in a desiccator, before the collection of blood from retro-orbital plexus for biochemical parameters. Blood collection was done in the morning. Euthanasia was performed by Cervical dislocation.

#### 3.10.1 Serum prolactin assay procedure

Prior to assay, all reagents and samples were allowed to reach room temperature (18 °C–26 °C) and gently mixed.

- The required number of antibody-coated microplate strips were placed into the holder.
- 25 μL of prolactin standards, controls, and rat serum samples were pipetted into the designated wells.
- 100 μL of enzyme-linked anti-prolactin HRP conjugate was added to each well.
- The plate was covered and incubated for 60 min at room temperature (18 °C–26 °C).
- The liquid was removed from all wells and each well was washed three times with 300 µL wash buffer, followed by blotting on absorbent paper.
- 100 μL of TMB substrate solution was added to all wells.
- The plate was incubated for 15 min at room temperature in the dark.
- 50 μL stop solution (usually 0.5 M sulphuric acid) was added to all wells and the plate was gently shaken to mix.
- The absorbance was measured at 450 nm using an ELISA plate reader within 15 min after adding the stop solution.

#### 3.10.2 Serum cortisol assay procedure

- All reagents, buffers, and serum samples were brought to room temperature before the assay.
- Standards and samples were analysed in duplicate to ensure accuracy.
- The required number of microplate strips were placed in the plate holder.

#### 3.10.3 Loading of well

Blank well: 100 µL of standard diluent was added.

Standard wells: 50 µL cortisol standard solution +50 µL streptavidin-HRP were added.

(Biotin-labelled antibody is pre-mixed in the standard preparation).

Sample wells: 40 µL serum sample +10 µL biotin-labelled cortisol antibody +50 µL streptavidin-HRP were added.

The plate was sealed and incubated at 37 °C for 60 min.

#### 3.10.4 Washing

After incubation, the plate was washed five times with wash buffer. Each wash consisted of filling the wells with washing buffer, allowing them to stand for 30 s, and discarding the liquid completely.

After the final wash, the plate was inverted and blotted on clean absorbent paper to remove residual buffer.

#### 3.10.5 Color development

50 µL chromogen reagent A was added to each well followed by 50 µL chromogen reagent B.

The plate was incubated for 10 min at 37 °C in the dark.

#### 3.10.6 Stop reaction

50 µL stop solution was added to each well.

The colour changed from blue to yellow immediately.

#### 3.10.7 Measurement

The absorbance was measured at 450 nm using a microplate ELISA reader within 10 min of adding the stop solution.

Cortisol concentrations were calculated by comparing the OD values with the standard calibration curve (Table 2).

**TABLE 2:** Serum prolactin and cortisol reagent calalogue.

Serum prolactin reagents	Serum cortisol reagents
Antibody-coated 96-well microplate	Pre-coated cortisol antibody microplate
Prolactin standards	Cortisol standard solutions
Anti-prolactin HRP conjugate	Biotin-labelled anti-cortisol antibody
Wash buffer concentrate	Streptavidin-HRP conjugate
TMB substrate solution	Standard diluent
Stop solution (acidic)	Wash buffer
Plate sealer	Chromogen reagent A (TMB)
​	Chromogen reagent B
​	Stop solution

### 3.11 Outcome variables

- Assessment of increase in pup body weight.
- Assessment of serum prolactin and cortisol levels in Wistar albino rats.
- Assessment of histomorphological alterations in mammary tissue of lactating rats, including increases in alveolar size, secretory material, and glycogen content.

### 3.12 Statistical analysis

One-way analysis of variance (ANOVA) followed by Bonferroni Tukey was used for statistical analysis.

## 4 Results

### 4.1 Result of HPTLC of the trial drug

The triplicate analysis at 254 nm showed four peaks in track 1, three peaks in track 2, and two peaks in track 3. An intense peak was observed at an Rf value of 0.03 in all three tracks, along with minor peaks at Rf values of 0.54 in track 1, 0.56 in track 2, and 0.05 in track 3 (Tables 3, 4).

**TABLE 3:** Result of physico-chemical evaluation.

Parameters	Result
Loss on drying	11.975%w/w
Ash value	10.467%w/w
Acid insoluble ash	3.7%w/w
Water soluble extractive	20%w/w
Alcohol soluble extractive	2.8%w/w

**TABLE 4:** Result of phytochemical analysis.

Test	Result
Alkaloids	+
Carbohydrates	+
Flavanoids	+
Glycosides	−
Phenol	+
Protein	+
Saponins	+
Tannin	+
Terpinoids	+

**FIGURE 1:**
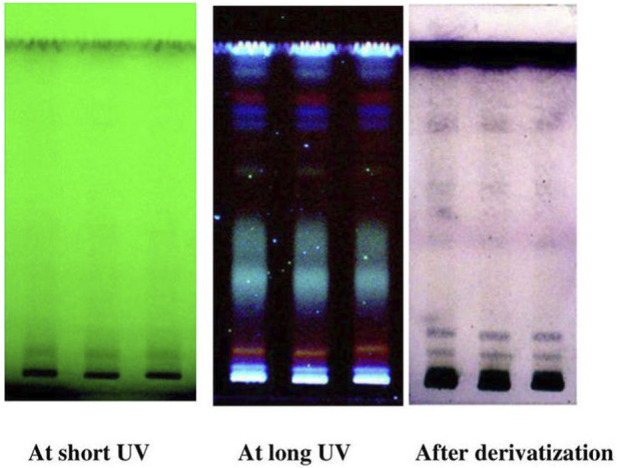
HPTLC documentation of trial drug.

At 366 nm, twelve peaks were observed in track 1, fourteen peaks in track 2, and eleven peaks in track 3. An intense peak was noted at an Rf value of 0.02 in track 1 and 0.03 in tracks 2 and 3. Minor peaks were observed at Rf values of 0.81 in track 1, 0.80 in track 2, and 0.81 in track 3 (Table 5; Figure 1).

**TABLE 5:** Result of heavy metal analysis.

Heavy metal	Result
Arsenic	0.28 ppm
Cadmium	BDL
Lead	6.74 ppm
Mercury	BDL

### 4.2 Result of *in-vivo* study

**GRAPH 1:**
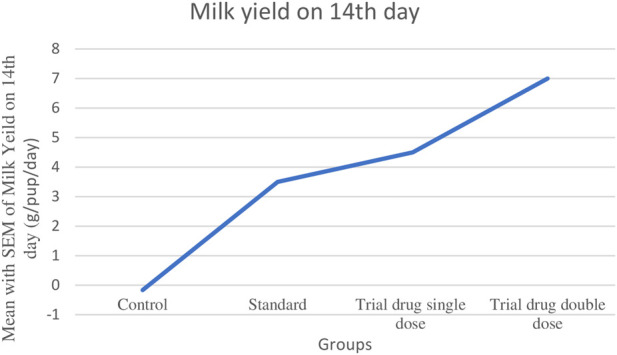
Mean with SEM of milk yield of on 14th day.

Milk yield on 14th day increased in Trial drug single dose group and Trial drug double dose group compared to Domperidone induced standard group and Control group but, it was statistically insignificant (Graph 1).

**GRAPH 2:**
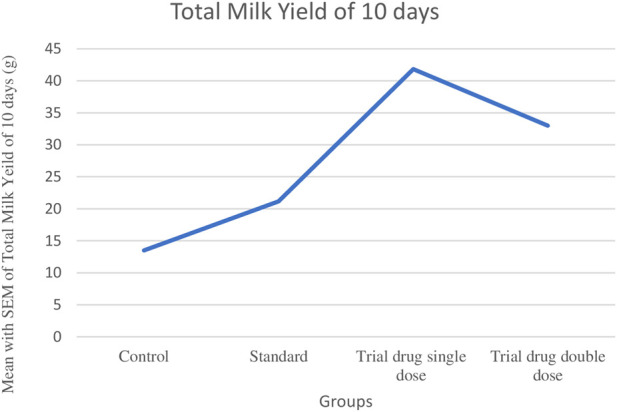
Mean with SEM of total milk yield of 10 days.

Milk yield of 10 days in Trial drug single dose group has increased significantly (*P value <0.05) compared to control group. Trial drug double dose has increased compared with Domperidone-induced standard group and control group but, it was statistically insignificant (Graph 2).

**GRAPH 3:**
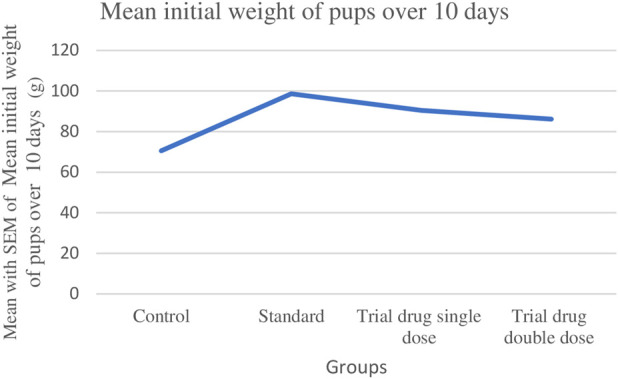
Mean with SEM of mean initial weight of pups over 10 days.

The mean initial weights of pups over 10 days of lactation increased in all three groups. Domperidone-induced standard group showed highest mean initial weights compared to control group, the observed values increased but, it was statistically insignificant (Graph 3).

**GRAPH 4:**
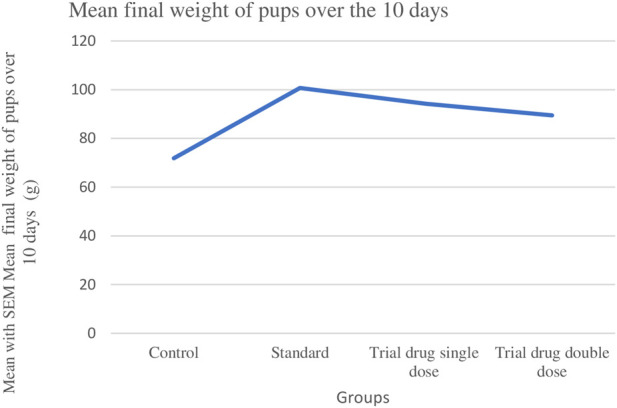
Mean with SEM of mean final weight of pups over 10 days.

The mean final weights of pups over 10 days of lactation increased in all three groups. Domperidone-induced standard group showed highest mean final weights compared to control group, the observed values increased but, it was statistically insignificant (Graph 4).

**GRAPH 5:**
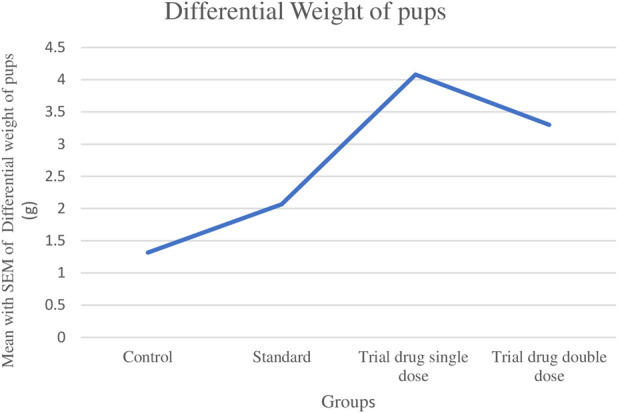
Mean with SEM of mean of differential weight of pups.

Differential weight of pups increased in all three groups. Trial drug single dose group showed highest differential weight of pups when compared with Domperidone induced standard group and control group the observed values increased but, it was statistically insignificant (Graph 5).

**GRAPH 6:**
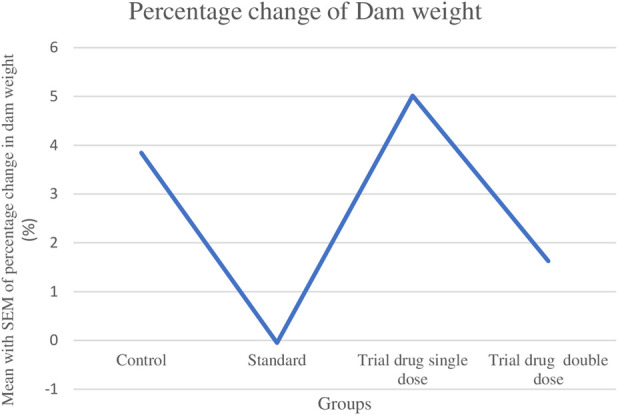
Percentage change of dam weight.

Percentage change of dam weight in Trial drug single dose group increased when compared to Domperidone induced standard group and control group but, it was statistically insignificant and decreased in Domperidone induced standard group and Trial drug double dose group (Graph 6).

### 4.3 Result of biochemical examination

**GRAPH 7:**
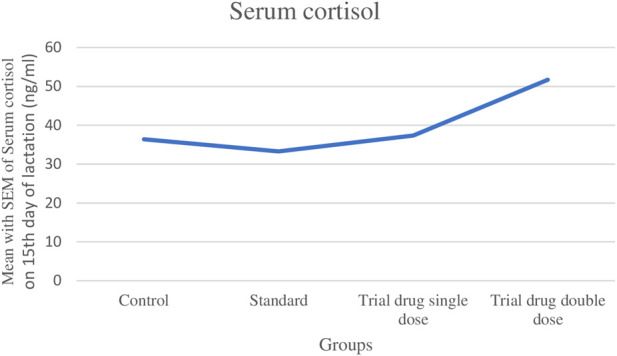
Mean with SEM of serum cortisol on 15th day of lactation.

Serum cortisol in Trial drug double dose group has increased significantly (**P < 0.01), compared to Domperidone induced standard group and control group. Trial drug single dose group increased and Domperidone induced standard group has decreased (Graph 7).

**GRAPH 8:**
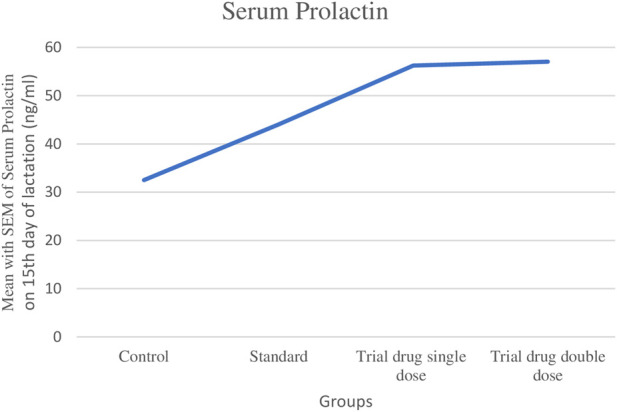
Mean with SEM of serum prolactin on 15th day of lactation.

Serum prolactin level in the trial drug single dose group and the trial drug double-dose group has increased significantly (****P < 0.0001) compared to control group. Trial drug single dose group has increased significantly (*P < 0.05) compared to Domperidone induced standard group. Trial drug double dose group increased significantly (**P < 0.01) compared to Domperidone induced standard group. Domperidone induced standard dose group increased significantly (*P < 0.05) compared with control (Table 6; Graph 8).

**TABLE 6:** Result of *in-vivo* study.

Parameters	Control	Standard		Trial drug single dose		Trial drug doubled dose	
	Mean ± SEM	Mean ± SEM	% Change	Mean ± SEM	% Change	Mean ± SEM	% Change
Milk yield on 14th day (g/pup/day)	0.1667 ± 0.9804	3.500 ± 1.996	1999.5↑ ns	4.500 ± 1.803	2,599.4↑ ns	7.000 ± 2.280	4,099.1↑ ns
Milk yield of 10 days (g)	13.50 ± 3.384	21.17 ± 6.052	56.81↑ ns	41.83 ± 6.89*	209.85↑	33.00 ± 10.09	144.4↑ ns
Mean initial weight of pups over 10 days (g)	70.53 ± 13.52	98.62 ± 11.64	207.7↑ ns	90.43 ± 10.47	147.18↑ ns	86.13 ± 21.89	115.38↑ ns
Mean final weight of pups over 10 days (g)	71.85 ± 13.72	100.7 ± 11.55 ns	40.15↑ ns	94.18 ± 11.00	31.07↑ ns	89.43 ± 22.90	24.46↑ ns
Differential weight of pups (g)	1.317 ± 0.3525	2.067 ± 0.6339	56.94↑ ns	4.0806 ± 1.806	210.02↑ ns	3.300 ± 1.009	150.56↑ ns
Percentage change of mother weight (%)	3.845 ± 1.300	−0.05000 ± 1.604	−98.69↓ ns	5.015 ± 2.281	30.42↑ ns	1.627 ± 1.518	−57.68↓ ns
Serum cortisol on 15th day of lactation (ng/mL)	36.39 ± 3.533	33.28 ± 4.565	−8.55↓ ns	37.34 ± 2.673	2.45↑ ns	51.71 ± 3.619**	42.009↑
Serum prolactin on 15th day of lactation (ng/mL)	32.50 ± 1.235	44.02 ± 2.848*	35.446↑	56.26 ± 3.073****	73.10↑	57.04 ± 2.740****	75.50↑

* P<0.05.

** P<0.01.

*** P<0.001.

**** P<0.0001.

ns, Non significant.

One-way analysis of variance (ANOVA) followed by Bonferroni Tukey was used for statistical analysis.

### 4.4 Result of histomorphological alteration of mammary tissue of lactating rats

**FIGURE 2:**
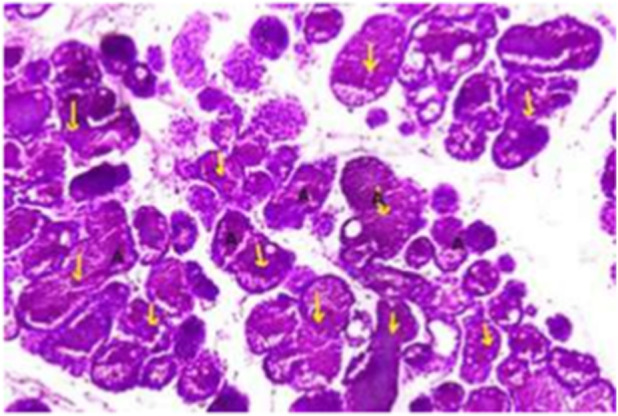
Control-the lumen of alveoli and ducts contain secretory material.

**FIGURE 3:**
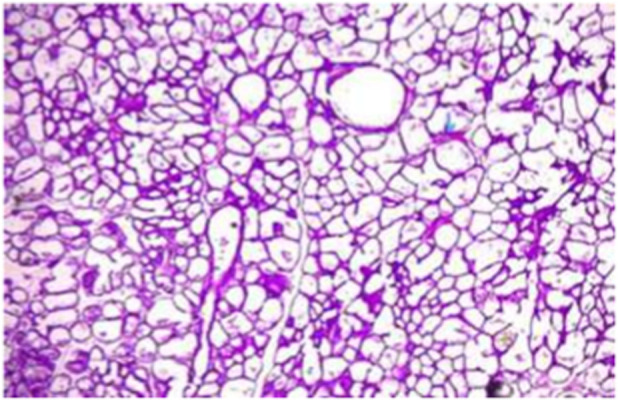
Control-mild presence of glycogen and proteoglycans are seen in the inter alveolar regions.

The mammary tissue revealed cuboidal epithelial lining of alveoli with luminal secretory material and mild PAS (Periodic acid Schiff) positive staining for glycogen and proteoglycans in the control group (Figures 2, 3).

**FIGURE 4:**
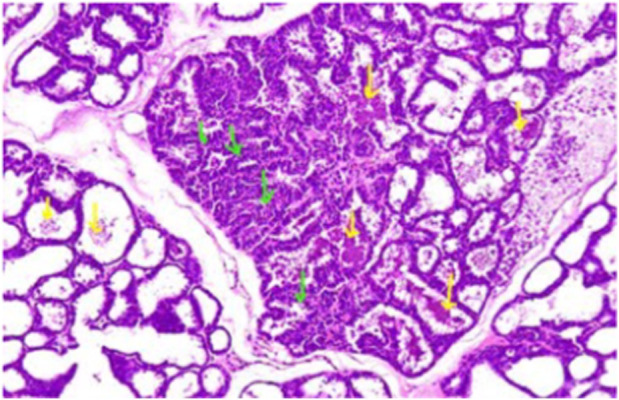
Standard-the lumen of alveoli and ducts contain secretory material.

**FIGURE 5:**
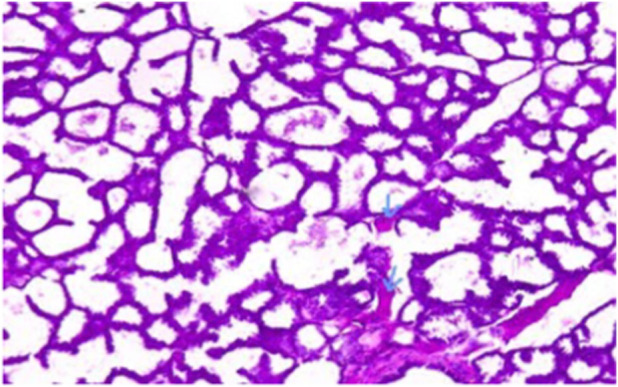
Standard-mild presence of glycogen and proteoglycans are seen in the inter alveolar regions.

The standard group showed a slight increase in alveoli with columnar epithelial lining, while secretory content and PAS staining remained comparable to the control, indicating mild galactagogue activity (Figures 4, 5).

**FIGURE 6:**
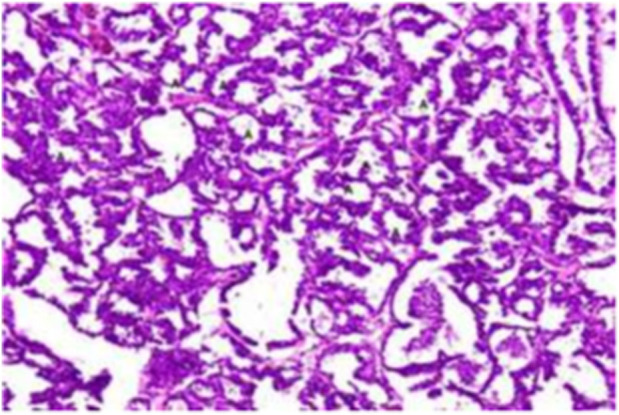
Trial drug single dose - increase in alveoli with hyperplasia (increase in number) and hypertrophy (increase in size) of cells in the alveoli.

**FIGURE 7:**
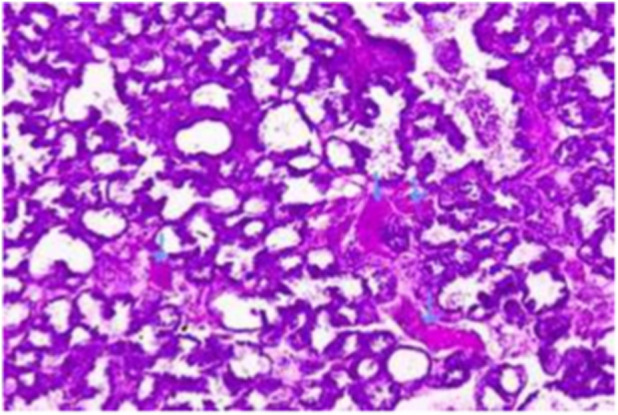
Trial drug single dose - presence of glycogen and proteoglycans.

The trial drug single dose group showed increased alveolar number and size and relatively more PAS-positive areas, suggesting mild increase (Figures 6, 7).

**FIGURE 8:**
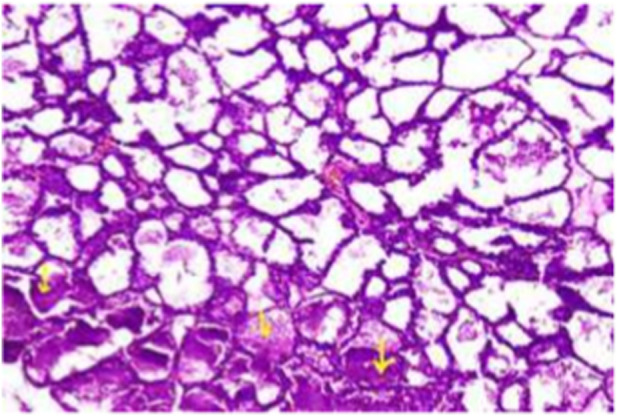
Trial drug double dose-the lumen of alveoli and ducts contain secretory material.

**FIGURE 9:**
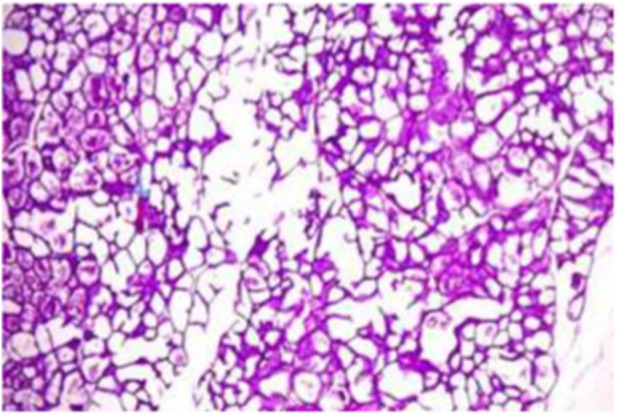
Trial drug double dose-mild presence of glycogen and proteoglycans are seen.

The double-dose trial group exhibited histological features similar to the control, with no notable increase in secretory material or PAS staining (Table 7; Figures 8, 9).

**TABLE 7:** Assessment of histomorphological alteration of mammary tissues of lactating rats.

Group	Secretory material	Increase in alveoli compared with control	Increase in size of alveoli	Increase in number of alveoli	PAS staining
C1	++	−	−	−	−
C2	++	−	−	−	+
S1	++	−	−	−	−
S2	++	+	−	−	+
T1-1	++	+	+	+	++
T1-2	++	−	−	−	+
T2-1	++	−	−	−	−
T2-2	++	−	−	−	+

C- control, S- standard, T1- trial drug single dose, trial drug double dose.

(−)- Absent, (+) – Mild, (++) Moderate.

## 5 Discussion

The findings of the present study demonstrate that *N. speciosum* rhizomes possess significant galactagogue activity, as evidenced by elevation of serum prolactin levels, which is a primary regulator of lactogenesis. Prolactin promotes differentiation of mammary alveolar epithelial cells and stimulates milk protein synthesis. The observed increase in prolactin in both treatment groups indicates that the test drug may act through modulation of the hypothalamic–pituitary axis, possibly by reducing dopaminergic inhibition. This mechanism is comparable to that of domperidone, a known dopamine antagonist used clinically to enhance lactation (Bruckmaier and Blum, 1998).

In addition to prolactin, serum cortisol levels were also elevated, particularly in the double-dose group. Cortisol plays a supportive role in lactation by facilitating mammary gland differentiation and synergizing with prolactin in milk synthesis. However, elevated cortisol may have complex physiological effects, including metabolic stress responses, which might explain why higher hormonal levels in the double-dose group did not proportionally translate into improved milk yield. Although higher prolactin levels exhibited in the double dose group and milk yield was lower compared to the single dose group. This may be due to the fact that Prolactin primarily regulates milk synthesis, whereas effective milk ejection depends on Oxytocin. Previous studies have shown that elevated prolactin does not always correspond to increased milk output. This suggests that lactation is influenced by multiple factors, including mammary gland function and effective milk ejection. Therefore, the reduced milk yield observed in the double dose group may be related to altered physiological responsiveness despite elevated prolactin levels (Özalkaya et al., 2018).

When compared to existing literature, the results align with previous reports on plant-derived galactagogues such as *Asparagus racemosus* and *Trigonella foenum-graecum*, which exert lactogenic effects through phytoestrogenic compounds and prolactin modulation (Pandey et al., 2005). Although specific studies on *N. speciosum* rhizomes are limited, the plant is known to contain bioactive constituents such as alkaloids, flavonoids- Quercetin (Ammar et al., 2025; Zhang et al., 2018) and Rutin (Badgujar and Bandivdekar, 2015), and phenolic compounds, which may contribute to endocrine modulation and tissue-level effects.

The findings suggest that the double dose demonstrates stronger galactagogue potential, although further studies with larger sample size and longer duration are required to confirm these effects.

## 6 Conclusion

The present study provides experimental evidence that śālūkaṃ (*N. speciosum* Willd rhizome) significantly elevates serum prolactin and cortisol levels, highlighting its influence on hormonal pathways associated with lactation. Both single and double dose groups were effective in enhancing these parameters when compared to the Domperidone-induced standard group. However, the double dose produced a more marked increase, indicating a clear dose-dependent effect. These findings suggest that higher dosing may offer improved hormonal stimulation, thereby supporting the potential of śālūkaṃ (*N. speciosum* Willd rhizome) as an effective natural galactagogue.

Therefore, śālūkaṃ (*N. speciosum* Willd rhizome) may contribute to natural lactation promoting therapy and could be developed into herbal formulations such as granules, tablets and powder for use in lactating mothers. Further studies are needed to optimize dosing and confirm clinical efficacy.

## Data Availability

The original contributions presented in the study are included in the article/supplementary material, further inquiries can be directed to the corresponding author.

## References

[B1] Ammar M. Russo G. L. Altamimi A. Altamimi M. Sabbah M. Al-Asmar A. (2025). *Moringa oleifera* supplementation as a natural galactagogue: a systematic review on its role in supporting milk volume and prolactin levels. Foods 14 (14), 2487. 10.3390/foods14142487 40724308 PMC12294722

[B2] Badgujar S. B. Bandivdekar A. H. (2015). Evaluation of lactogenic activity of an aqueous extract of *cyperus* Linn. J. Ethnopharmacol. 163, 39–42. 10.1016/j.jep.2015.01.019 25625349

[B3] Bora D. Mehmud S. Das K. K. Medhi H. (2016). Report on folklore medicinal plants used for female health care in Assam (India). Int. J. Herb. Med. 4 (6), 4–13.

[B4] Bruckmaier R. M. Blum J. W. (1998). Oxytocin release and milk removal in ruminants. J. Dairy Sci. 81 (4), 939–949. 10.3168/jds.S0022-0302(98)75654-1 9594382

[B5] Charan J. Kantharia N. D. (2013). How to calculate sample size in animal studies. J. Pharmacol. Pharmacother. 4 (4), 303–306. 10.4103/0976-500X.119726 24250214 PMC3826013

[B6] Festing M. F. W. Altman D. G. (2002). Guidelines for the design and statistical analysis of experiments using laboratory animals. ILAR J. 43 (4), 244–258. 10.1093/ilar.43.4.244 12391400

[B7] Hosseinzadeh H. Tafaghodi M. Mosavi M. J. Taghiabadi E. (2013). Effect of aqueous and ethanolic extracts of *Nigella sativa* seeds on milk production in rats. J. Acupunct. Meridian Stud. 6 (1), 18–23. 10.1016/j.jams.2012.07.019 23433051

[B8] Kumar R. Gupta V. (2015). Evaluation of lactogenic activity of an aqueous extract of *Cyperus rotundus* Linn. rhizome. J. Ethnopharmacol. 159, 130–136. 10.1016/j.jep.2015.01.019 25625349

[B9] Lajuna L. Sriyanti C. S. (2025). The influence of psychological factors on breast milk production among breastfeeding mothers. Sci. Midwifery 13 (2), 126–132.

[B10] Lin M. Wang N. Yao B. Zhong Y. Lin Y. You T. (2018). Quercetin improves postpartum hypogalactia in milk-deficient mice via stimulating prolactin production in the pituitary gland. J. Cell Physiol. 233 (8), 6238–6248. 10.1002/ptr.6079 29671937

[B11] Machado K. G. Balen A. H. Torsoni M. A. (2014). Pharmacological overview of galactogogues. J. Pharm. Pharm. Sci. 17 (4), 552–561.

[B12] Mukherjee P. K. Mukherjee D. Maji A. K. Rai S. Heinrich M. (2009). The sacred lotus (*Nelumbo nucifera*)—phytochemical and therapeutic profile. J. Pharm. Pharmacol. 61 (4), 407–422. 10.1211/jpp/61.04.0001 19298686

[B13] Murthy K. R. S. (2005). Bhavaprakasha of bhavamisra. 3rd ed. (Varanasi: Chaukhambha Vishvabharati), 2, 667.

[B14] Özalkaya E. Aslandoğdu Z. Özkoral A. Topcuoğlu S. Karatekin G. (2018). Effect of a galactagogue herbal tea on breast milk production and prolactin secretion by mothers of preterm babies. Niger. J. Clin. Pract. 21 (1), 38–42. 10.4103/1119-3077.224788 29411721

[B15] Pandey S. K. Sahay A. Pandey R. S. Tripathi Y. B. (2005). Effect of *Asparagus racemosus* on lactation. Indian J. Pharmacol. 37 (4), 241–246.

[B16] Piccolo O. Woo Kinshella M. L. Salimu S. Vidler M. Banda M. Dube Q. (2022). Healthcare worker perspectives on mothers’ insufficient milk supply in Malawi. Int. Breastfeed. J. 17, 18. 10.1186/s13006-022-00460-1 35197105 PMC8867656

[B17] Rahman M. M. Islam M. S. Hossain M. A. Uddin M. G. (2019). Pharmacognostic and acute toxicity study of the rhizome of *Nymphaea lotus* L. Asian J. Med. Biol. Res. 5 (2), 138–145.

[B18] Samhita S. S. (2012). in Madhyama Khanda, chapter 6, verse 1. Editor Murthy K. R. S. (Varanasi: Chaukhambha Orientalia).

[B19] Sampson D. A. Jansen G. R. (1984). Measurement of milk yield in the lactating rat from pup weight and weight gain. J. Pediatr. Gastroenterol. Nutr. 3 (4), 613–617. 10.1097/00005176-198409000-00023 6481568

[B20] Tiwari M. Singh R. H. (2012). Ethnomedicinal practices among the tribes of Lalganj block of district Mirzapur, Uttar Pradesh. Life Sci. Leafl. 10, 84–99.

[B21] Zhang Y. Liu X. Wang Y. (2018). Quercetin improves postpartum hypogalactia in mice by stimulating prolactin production and prolactin receptor expression. J. Ethnopharmacol. 217, 97–105.

